# Neutron capture-enhanced proton beam therapy outside the irradiation field: in vitro experiments

**DOI:** 10.1186/s13104-025-07362-5

**Published:** 2025-07-15

**Authors:** Shintaro Shiba, Takahiro Shimo, Masashi Yamanaka, Kazuki Matsumoto, Akihiro Yamano, Kazunori Nitta, Makoto Sakai, Tatsuya Ohno, Koichi Tokuuye, Motoko Omura

**Affiliations:** 1 Department of Radiation Oncology, Shonan Kamakura General Hospital, 1370-1, Okamoto, 247-8533 Kamakura, Kanagawa Japan; 2 Department of Radiation Oncology, Gunma University Graduate School of Medicine, Gunma, Japan; 3 Radiological Research Division, Shonan Research Institute of Innovative Medicine, Shonan Kamakura General Hospital, Kanagawa, Japan; 4 Department of Medical Physics, Shonan Kamakura General Hospital, Kanagawa, Japan

**Keywords:** Proton beam therapy, Neutron capture-enhanced proton beam therapy, Radiotherapy, Boron neutron capture reaction, Boron neutron capture therapy, Boronophenylalanine

## Abstract

**Objective:**

To evaluate the cell-killing effects of neutron capture-enhanced particle therapy (NCEPT) outside the proton beam irradiation (PBI) field.

**Results:**

Human osteosarcoma cells (MG63) were divided into control and NCEPT groups. Four hours before irradiation, the NCEPT group was exposed to ^10^B-boronophenylalanine. PBI was performed by targeting a gel bolus, with tissue culture plates placed outside the PBI field adjacent to the gel bolus. The bolus was irradiated with 12, 36, or 72 Gy. Cell survival fractions were calculated for wells adjacent to the bolus. Furthermore, the neutron fluence generated by PBI in the tissue culture plates was calculated using a Monte Carlo simulation. After 12, 36, and 72 Gy bolus irradiation, the cell survival rates in the control group were 103%, 95%, and 95%, respectively, whereas those in the NCEPT group were 84%, 75%, and 51%, respectively. The cell survival fraction in the NCEPT group was significantly lower than that in the control group (*P* < 0.01). The mean neutron fluence in the wells adjacent to the bolus was 1.24 × 10^8^ (1/cm^2^) at 72 Gy irradiation. Although NCEPT enhanced cell-killing outside the PBI field, the magnitude of this effect was modest.

## Introduction

Proton beam therapy (PBT) is increasingly used because of its superior dose localization compared with X-ray radiotherapy, allowing for higher-dose administration and improved clinical outcomes [1–3]. Chemotherapeutic agents are often used as sensitizers to enhance the treatment efficacy. Concurrent chemotherapy with PBT is used to prevent distant metastasis and increase local effects; however, it also increases toxicity [4, 5]. An ideal sensitizer for local effects would increase therapeutic efficacy without contributing to toxicity. In this regard, chemotherapy is not an optimal sensitizer for local effects, owing to its associated toxicity.

Neutron capture-enhanced particle therapy (NCEPT) has recently been proposed as an alternative approach [6]. This method utilizes neutrons generated by nuclear reactions during irradiation with particle beams such as proton and carbon ion beams to induce neutron capture reactions and achieve a sensitizing effect. Boronophenylalanine (BPA), a boron agent used in the clinical practice of boron neutron capture therapy (BNCT), is selectively taken up by tumors through transporters such as L-type amino acid transporter 1 (LAT1), which is specifically expressed in tumors [7]. Although BPA is taken up in small amounts by healthy cells, its selective accumulation in tumors makes it a promising candidate for targeted therapy. Neutrons and boron undergo a boron neutron capture reaction (BNCR), producing alpha particles and lithium ions [^10^B (n, α) ^7^Li]. These particles exhibit high linear energy transfer (LET) and have a range of approximately 5–9 μm, which is comparable to the diameter of tumor cells. Consequently, they act selectively on cells that take up boron and exert a strong cell-killing effect [8]. Therefore, the use of BPA in PBT may enhance tumor sensitization, while minimizing its effects on healthy tissues. This feature may be useful for improving the therapeutic effect on radioresistant tumors such as sarcomas, which have a limited response to low-LET radiation of PBT. Furthermore, because the neutrons generated by proton beam irradiation (PBI) reach outside the irradiation field, NCEPT using these neutrons may be effective against nearby infiltrates and micrometastatic lesions outside the irradiation field. To date, in vitro experiments have demonstrated the sensitizing effects of NCEPT. Studies using proton beams by our group and carbon/helium ion beams by other groups have confirmed these findings [9, 10]. However, the efficacy of NCEPT with PBI against nearby infiltrates and micrometastatic lesions outside of the irradiation field has not been sufficiently explored.

This study evaluated the cell-killing effects of NCEPT outside the irradiation field of PBI. In addition, to examine the contribution of neutrons to the cell-killing effect of NCEPT, the neutron fluence outside the irradiation field generated by the PBI was calculated using a Monte Carlo simulation.

## Materials and methods

### Cell culture

The human osteosarcoma cell line (MG63) was obtained from the Japanese Collection of Research Bioresources Cell Bank (JCRB). The cells were cultured and maintained in Dulbecco’s modified Eagle’s medium supplemented with 10% heat-inactivated fetal bovine serum and 1% penicillin-streptomycin at 37 °C in a humidified atmosphere with 5% CO_2_. The medium and serum were purchased from Fujifilm Wako Pure Chemical Corporation (Tokyo, Japan). Cells were passaged preconfluent, and within 20 passages after purchase from JCRB, were used for all experiments.

### Boron compounds

^10^B-enriched BPA (> 95% ^10^B) was provided by Stella Pharma Corporation (Osaka, Japan). A 10% molar excess of fructose (Fujifilm Wako Pure Chemical Corporation, Osaka, Japan) was added to the medium to increase the solubility of BPA. The boron concentration in the medium was measured using inductively coupled plasma optical emission spectrometry (ICP-OES) (Spectrogreen, Hitachi High-Tech, Tokyo, Japan) and was controlled at 80 ppm.

### Monte carlo simulations

**Fig. 1:**
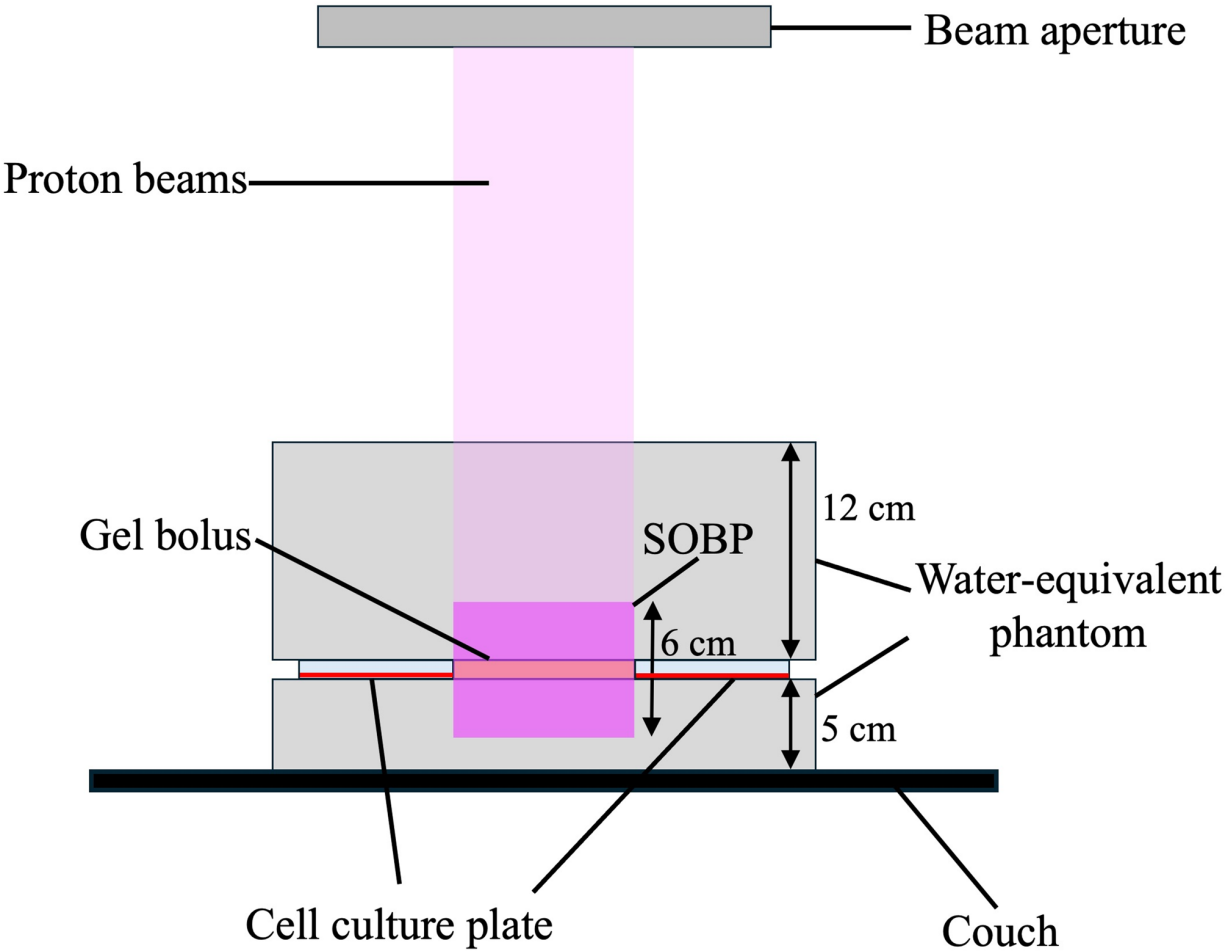
Schematic diagram of the experimental configuration of irradiation SOBP, spread-out Bragg peak

Monte Carlo simulations were performed using a Particle and Heavy Ion Transport code system (version 3.32) to determine the proton and thermal neutron fluence distributions [11]. Dose calculations were performed using the PBT system (PROBEAT-M1; Hitachi, Tokyo, Japan) at Shonan Kamakura General Hospital (SKGH). Previous studies have reported Monte Carlo simulations using beam data and dosimetry of the PBT system [9]. In Monte Carlo simulations, 5.0 × 10⁸ histories were simulated to ensure that the statistical uncertainty remained below 1%. Variance reduction techniques were not applied because the computational cost was acceptable and sufficient statistical precision was attained by simulating a large number of particle histories. Simulations were performed using proton beams with the following conditions: Spot-scanning methods; a spread-out of Bragg peak (SOBP) width, 6 cm; energy range, 139.0-154.5 MeV; a spot spacing, 5 mm; a field size, 12.5 × 9.6 cm (corresponding to gel bolus size). A gel bolus (15 × 9.8 × 2 cm) was positioned at 12 cm, which is the center of the SOBP, below the surface of a water-equivalent phantom (RW3, PTW, Freiburg, Germany) measuring 30 × 30 × 17 cm (Fig. 1). Six-well tissue culture plates were placed next to either side of the gel bolus, outside the PBI field. The phantom, bolus, and cell culture plate materials were replicated precisely to reflect the experimental conditions. The thermal neutrons were defined as those with energies of ≤ 0.6 eV [12].

### Proton beam irradiation

The clinically used PBT system at SKGH was used in this study. PBI was performed at SKGH with the following conditions: Spot-scanning methods; a SOBP width, 6 cm; energy range, 139.0-154.5 MeV; a spot spacing, 5 mm; a field size, 12.5 × 9.6 cm. PBI was performed by placing phantoms at the proximal 12 cm and distal 5 cm of a 6-well tissue culture plate and a gel bolus. The gel bolus was positioned at the center of the SOBP with its edges at a proton beam dose of 50% isodose. Six-well tissue culture plates were placed adjacent to each side of the gel bolus outside the PBI field (Fig. 1). The physical irradiation dose was calculated by using a treatment planning system (VQA, Hitachi, Japan). The bolus was irradiated with different doses (12, 36, and 72 Gy).

### Irradiation experimental procedures and clonogenic cell survival assay

Irradiation and clonogenic cell survival assays were performed as previously described [9]. Briefly, wells adjacent to the bolus were used to evaluate cell survival. Cells were seeded in six-well tissue culture plates 24–48 h before PBI treatment. Four hours before PBI, cells in the NCEPT group were exposed to BPA (80 ppm boron), whereas only the medium was replaced in the control group. The plates were placed adjacent to the bolus for the PBI (outside the irradiation field) (Fig. 1). After PBI, the BPA-containing media from the NCEPT and control groups were removed. Cells in both groups were washed with PBS and the medium was replaced with BPA-free medium. After incubation for 10–14 days, the cells were fixed with methanol and stained with crystal violet. The cell surviving fractions were normalized to the surviving fraction in the absence of irradiation (control) for each group.

### Intracellular boron concentration

Intracellular boron concentration was measured as described previously [9]. Cells were exposed to BPA (80 ppm boron) for 4 h. After exposure to BPA, the cell membrane was disrupted by surfactant after processes such as washing and centrifugation, and the intracellular boron concentration was measured using ICP-OES.

### Statistical analysis

All experiments were performed with at least three replicates. Data obtained from three independent experiments are expressed as the mean ± standard deviation. Differences were statistically analyzed using the Wilcoxon signed-rank test. Statistical significance was set at *P* < 0.05.

## Results

### Neutron fluence and intracellular boron concentration

**Fig. 2:**
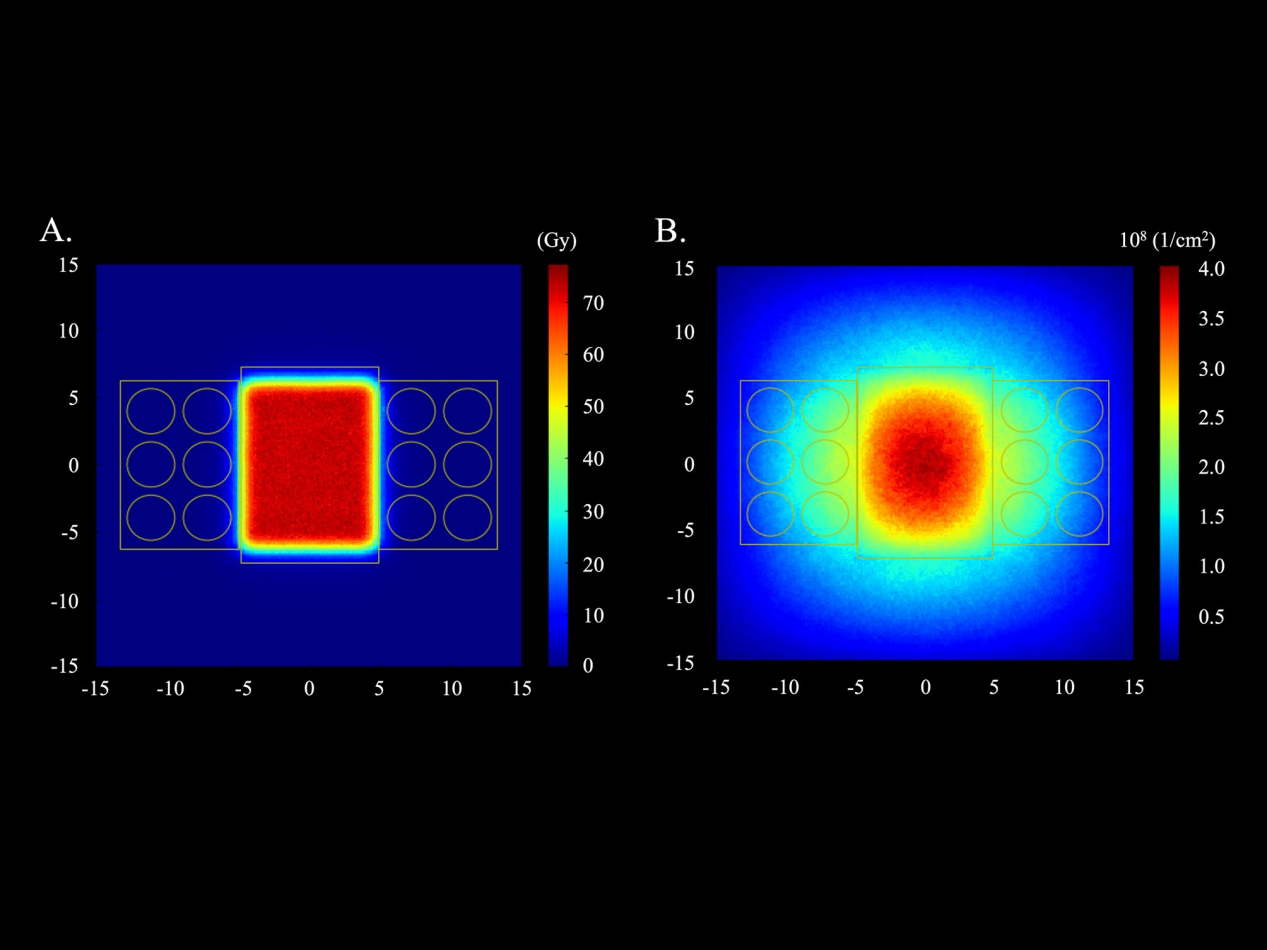
Fluence distributions of the spread-out of Bragg peak central cross section calculated using Monte Carlo simulations at 72 Gy irradiation. The yellow squares show the wells used in the clonogenic cell survival assay. (**A**) Proton fluence distribution. (**B**) Thermal neutron fluence distribution

The proton and thermal neutron fluence distributions calculated using Monte Carlo simulations with 72 Gy irradiation are shown in Fig. 2. The mean neutron fluence in the wells adjacent to the bolus of the six-well tissue culture plates was 1.24 (range: 1.18–1.35) (10^8^ [1/cm^2^]) at 72 Gy irradiation. The mean intracellular boron concentration was 392 (range: 317–506) ppm.

### Cell survival curve

**Fig. 3:**
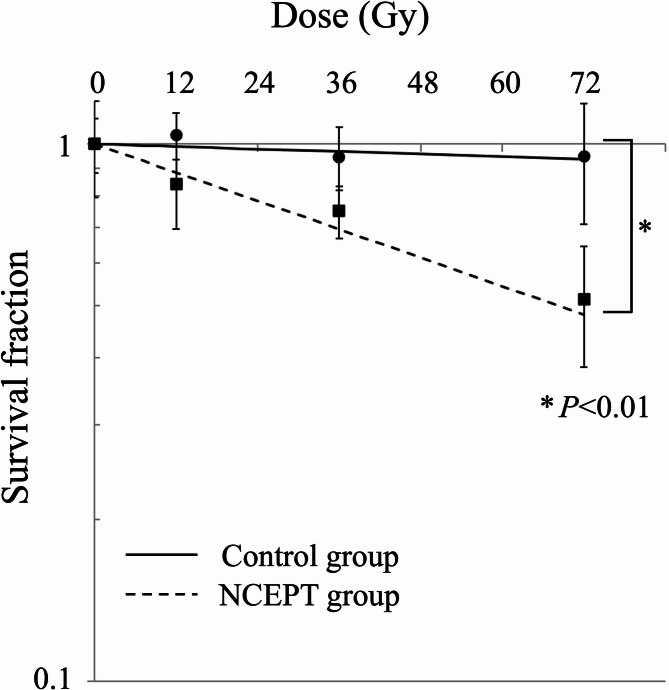
Survival curves of MG63 cells. Control group (solid line), NCEPT group (dashed line). Data are presented as the mean ± standard deviation and fitted to the linear-quadratic model. The vertical axis represents the logarithmic cell survival fraction, and the horizontal axis represents the physical dose of PBT irradiated to the gel bolus. * *P* < 0.01 NCEPT, neutron capture enhanced particle therapy

The survival curves for the control and NCEPT groups under different irradiation schemes are shown in Fig. 3. The horizontal axis in Fig. 3 shows the physical dose irradiated to the gel bolus calculated using the PBT treatment planning system, which does not include the dose generated by BNCR. The cell survival fractions in the control group after 12, 36, and 72 Gy bolus irradiations were 103%, 95%, and 95%, respectively, whereas those in the NCEPT group were 84%, 75%, and 51%, respectively. The cell survival fraction in the NCEPT group was significantly lower than that in the control group (*P* < 0.01).

## Discussion

Monte Carlo simulations and in vitro experiments revealed that neutrons generated by proton irradiation spread outside the irradiated field and induced BNCR, resulting in a cell-killing effect. Our findings suggest that NCEPT may be effective against unexpected microinvasions and metastases near fields irradiated with proton beams, although its cell-killing effects are limited and may not be clinically significant.

A previous study showed that NCEPT, using carbon and helium ion beams, exhibited a cell-killing effect outside the irradiation field [10]. Unlike NCEPT, which uses proton beams in the present study, a higher cell-killing effect was observed at low physical doses. The cell survival rate outside the irradiated field after 6 Gy irradiation of the target (flask filled with culture medium) was less than 10%, which was considerably different from the 51% cell survival rate at 72 Gy in the present study. One reason for this difference may be the large differences in the irradiation systems. This study used the spot-scanning method, whereas the previous study used the passive scattering method, in which the beam passes through beam-shaping assemblies, such as ridge filters and collimators. In the passive scattering method, many objects are present on the beamline from which the neutrons are generated. In a previously reported NCEPT study, we believe that these neutrons were also utilized for BNCR, resulting in a higher cell-killing effect than that observed in this study.

This study explored the potential clinical applications of NCEPT. However, the doses used were extremely high. At a dose of 12 Gy, which may be used clinically, the cell-killing effect outside the irradiation field was limited. Even at 72 Gy, the cell survival rate was 51%, which is equivalent to approximately 2 Gy of PBI alone [13]. Therefore, the effects of NCEPT on nearby infiltrates and micro-metastatic lesions outside the irradiation field should not be overestimated in clinical practice. In contrast, the results of this study suggest that the effect on healthy tissues outside the PBI field on the lateral side is negligible in clinical practice. In the clinical application of BNCT, BPA uptake in normal cells is thought to be less than 1/2.5 (tumor/normal tissue boron concentration ratio > 2.5) of that in tumor cells [14]; therefore, NCEPT-induced toxicity is expected to be minimal.

### Limitations

This study had certain limitations. First, a single cell line was used. However, in previous NCEPT experiments that confirmed an increased cell-killing effect within the irradiation field, multiple cell lines were used, and the mechanism by which BNCR occurred remained consistent; only the neutron dose differed [9]. Therefore, it is reasonable to assume that the cytotoxic effects observed in this study can be reproduced in other cell lines. Second, the experiment was conducted using only proton beams and the spot-scanning method. Given the differences in the results compared with previous studies using carbon and helium ion beams, it would be valuable to compare the outcomes under matched conditions, such as the particle type and beam supply method. Third, only the neutron fluence was calculated in this study; an accurate dose calculation for NCEPT was unavailable. The accurate dose calculation of NCEPT, such as alpha particles and lithium ions generated by BNCR, is one of the next challenges that we are focusing on.

## Conclusion

An increase in the cell-killing effect of NCEPT was observed outside the irradiation field of PBI. However, the effect outside the irradiated field was low, and its clinical significance was limited.

## Data Availability

The datasets generated for this study are available to the corresponding author upon request.
